# “…[T]his is What We are Missing”: The Value of Communicating Infant Feeding Information Across Three Generations of African American Women

**DOI:** 10.1177/0890334421995078

**Published:** 2021-02-25

**Authors:** Alexis L. Woods Barr, Deborah A. Austin, Jacquana L. Smith, Ellen J. Schafer

**Affiliations:** 1 41474 2331 Department of Maternal and Child Health, Carolina Global Breastfeeding Institute, Gillings School of Global Public Health, University of North Carolina at Chapel Hill, USA; 2 REACHUP, Inc, Tampa, FL, USA; 3 1791 Department of Community and Environmental Health, College of Health Sciences, Boise State University, Boise, ID, USA

**Keywords:** African America, Black Feminist Theory, breastfeeding, breastfeeding experience, breastfeeding knowledge, breastfeeding support, cultural norms, infant feeding patterns, intergenerational, social support

## Abstract

**Background:**

Breast/Chestfeeding remains a public health issue for African Americans, and increased rates would mitigate many health disparities, thus promoting health equity.

**Research Aims:**

To explore the interplay of generational familial roles and meaning (or value) ascribed to communicating infant feeding information across three generations.

**Method:**

This prospective, cross-sectional qualitative study used an asset-driven approach and was guided by Black Feminist Thought and Symbolic Interactionism. African American women (*N* = 35; 15 family triads/dyads), residing in the southeastern United States were interviewed. Data were analyzed using thematic analysis.

**Results:**

The older two generations described their role using assertive yet nurturing terms, while the younger generation carefully discussed the flexibility between their familial roles. Emergent themes described the meaning each generation attributed to communicating infant feeding information: “My Responsibility,” “Comforting,” “Bonding Experience,” “She Cared,” and “Gained Wisdom.”

**Conclusions:**

Our findings have potential to contribute to achieving health equity in African American families. Future breast/chestfeeding promotion efforts may benefit from reframing the current approach to including protection language and not solely support language. Lactation professionals should further recognize and support strengths and resource-richness of intergenerational infant feeding communication within African American families using strength-based, empowerment-oriented, and ethnically sensitive approaches.

Breast/chestfeeding is associated with desired health outcomes for infants and mothers/birthing parents. Although below the national average, the majority (74%) of non-Hispanic Black/African American mothers in the United States initiate breastfeeding (Centers for Disease Control and Prevention, 2019), and stand to gain a great deal in terms of their lifelong health from improved breastfeeding behaviors. African Americans disproportionately suffer from various health disparities, including the highest rates of prematurity, low birthweight, diabetes mellitus, breast and ovarian cancer, infant mortality, and maternal mortality (Bartick et al., 2017). Breastfeeding has the potential to mitigate resulting poor birth outcomes and reduce risks of infant and maternal morbidity and mortality (American Academy of Pediatrics, 2012). Hence, breastfeeding is a vital public health issue for African Americans, and increasing their rates is essential to eliminating these health disparities, and thus promoting health equity (Anstey et al., 2017).

Health communication strategies are used in clinical settings to address health disparities (Hovick et al., 2015). However, because of the history of discrimination and medical mistrust, African Americans tend to rely on their extended kinship networks for health-related information (Pullen et al., 2015). Often, health communication occurs in African American families through oral histories, storytelling, and narratives rooted in African American culture (Fabius, 2016). These informal methods of passing information from generation to generation have many purposes, including an emancipatory function to counter dominant ideologies, and to teach younger generations about the resilience and perseverance unique to the African American experience (Fabius, 2016). This experience dates back to the historical narratives from chattel slavery that include anti-Black racism, involuntary breeding, sexual assault, wet-nursing, child abduction, forced sterilization, and maternal vilification, and the effects of this legacy are still ongoing (Fabius, 2016; Gatison, 2017; Roberts, 1997; West & Knight, 2017). “Stories and rituals are symbolic links to the past, performed in the present. Thus, they may be regarded as a means for understanding family communication as an oral tradition” (Jorgenson & Bochner, 2004, p. 518). Family infant feeding communication, and the quality and availability of social support influences breastfeeding outcomes for African Americans (DeVane-Johnson et al., 2017).

In African American families, elders are entrusted keepers of communal knowledge, and hailed as the wisest, most respected members of the family (McLoyd et al., 2019). They play an important role in preserving their cultural beliefs and family values (including family reciprocity, sense of duty, and group survival). Other aspects of their role are passing down communication values and ideals, which are foundational for intergenerational support in their flexible family system (McLoyd et al., 2019). Therefore, African American mothers tend to consult their own mother and maternal grandmother for parenting guidance and advice rather than healthcare providers (Grassley et al., 2012). Grandmothers (an infant’s grandmother and great-grandmother) play a critical role in infant feeding decisions and may act as a postpartum breastfeeding advocate (Grassley & Eschiti, 2011). Furthermore, researchers have postulated that since many grandmothers in the United States may lack breastfeeding knowledge and experience, this may influence the support and advice they provide to new birthing parents (Grassley et al., 2012). In fact, a grandmother’s lack of understanding (or misunderstanding) of current breastfeeding recommendations may influence a parent’s breastfeeding self-efficacy, supply, and overall success (Grassley et al., 2012). However, strength-based literature has suggested that although family feeding history may influence the expression of social support, positive social support exists within African American families (Woods Barr et al., in press).

**Key Messages**

- We explored the interplay of generational familial roles and meaning (or value) that African American female family members ascribe to sharing infant feeding information across three generations.
- Concerning the meaning of shared infant feeding information, older generations described their moral responsibility, the middle generation expressed comfort and bonding, and the younger generation reported their trust in older generations.
- Lactation professionals should recognize the value of multigenerational oral traditions and consider including protection language in addition to support language when including elders into infant feeding conversations.

Given that African Americans experience breastfeeding disparities and health disparities, understanding the key sociocultural contexts in which infant feeding is communicated is important. An understanding of the meaning African Americans give to shared infant feeding information within their family is necessary (Peritore, 2016). African Americans are often bombarded with messages, images, and stereotypes of “good” motherhood from multiple channels; however, these messages may conflict with the complex relationships African Americans have with their bodies, families, and communities, as a result of their historically negative reproductive experiences in America (Johnson et al., 2015). The authors posit that the socially-constructed meanings of infant feeding information, passed down from generation to generation by a mother’s own mother and/or maternal grandmother, helps to shape feeding practices among the younger generation. To better understand the meaning of sharing infant feeding information, it is important to consider how each generation defines and navigates their familial roles. These are missing components in the comprehensive study of family health and infant feeding communication. Therefore, the aim of this study was to explore the interplay of generational familial roles and meaning (value) ascribed to communicating infant feeding information across three generations of African American women.

## Theoretical Frameworks

Black Feminist Thought (BFT) and Symbolic Interactionism informed and guided this research. BFT provides a lens to view the intersectional experiences of Black women and the ways in which they interact with society (Collins, 2000). As a theoretical framework, BFT strives to change the narrative of Black women, highlight their compounding forms of oppression, and express the value of culture in their lives (Collins, 2009). Collins (2009) supported the idea that self-definition permits Black women to rid themselves of the negative images and assumptions created by white society, as an act of empowerment that counteracts marginalization. Symbolic Interactionism was chosen alongside BFT because Symbolic Interactionism is a communication theory of human behavior (Faules & Alexander, 1978). Symbolic Interactionism provided a framework for making meaning of lived experiences from the actor’s viewpoint. *Meaning* is one of the core essentials for understanding human behavior, interactions, and social processes. Symbolic interactionists have suggested that to fully understand a person’s social processes, one needs to understand the meanings that an individual places on experiences within a specific context (Chenitz & Swanson, 1986; Morris, 1977). Both theories emphasize a person’s lived experience, which includes their internal human behavior, the concept of meaning perceived by them, and understanding context from their perspective (Jeon, 2004).

## Method

### Design

Based on the gaps identified, this study adopted a prospective, cross-sectional qualitative research design using an asset-driven approach, centering African American women’s voices and lived experiences (Brown, 2012). Compared to other research methods, qualitative research is unique because it allows the researcher to capture narratives, feelings, and thoughts. This study was approved by the University of South Florida Institutional Review Board.

### Setting

Women living in the Southeastern region of the United States tend to breastfeed less often than women living in other regions, regardless of sociodemographic characteristics (Anstey et al., 2017). Additionally, women living in this region (particularly Black women) tend to experience disproportionately high rates of cesarean sections (many of which are medically unnecessary) (Centers for Disease Control and Prevention, 2018). In addition to several short-term and long-term health risks for mothers and their infants, cesarean sections are associated with lowered breastfeeding rates (Chen et al., 2018).

### Sample

A sample of African American women (*N* = 35; 15 family triads/dyads) were recruited using purposive and snowball sampling (Patton, 2002). Family triads included the youngest adult generation (G3), her mother/mother figure (G2), and her maternal grandmother/grandmother figure (G1). Family dyads consisted of G3 and G2. Grandmother and mother figures (or other mothers) consisted of aunts, sisters, cousins, and stepmothers who were responsible for raising G3 (Collins, 2005). All participants were adult women who self-identified as African American, Black, Colored, or Negro, were a part of a family where at least two generations were willing to participate in the study, and at least one generation in each family resided in the southeastern United States. Additionally, the youngest adult generation needed to have had at least one child that they breastfed for 3 months or more, and the child was 5 years or younger at the time of the study. Families were excluded if at least one woman in the dyad/triad reported not being in active communication with the other woman/women in the family. Participants were recruited until a sample size adequate for qualitative research and thematic saturation was reached (Morse, 1994).

### Data Collection

From February–March 2019, in-person and telephone interviews were conducted with African American women living in the southeastern United States. The first author (A. W. B.) is an African American female doctoral trained researcher who was the project leader and sole data collector for this project. Her identity mediated access to the study sample and the depth of information that each participant shared with her. Throughout the data, participants used words and phrases like “we,” “us,” and “you know,” reflecting the race concordance between the first author and participants. This is a methodological strength of the study.

A.W. B. obtained informed consent immediately before conducting the interview. For both in-person and telephone interviews, participants had the opportunity to ask questions about the informed consent prior to beginning the interview. In each instance, interviews were audio recorded. All recorded and written data were kept confidential. Measures were taken to protect the storage of research-related records on a secure research server to which only A. W. B. had access.

A.W. B. developed and pilot tested two interview guides: one for older generations (G1s/G2s), and one for the youngest generation (G3s). She used loosely structured interviews to engage with participants using a small list of core questions and probes (ensuring similar data were collected from each participant), while also allowing them to tell their stories in their own way (Davis & Craven, 2016). Considering the sensitivity of discussed topics, A. W. B. interviewed participants in a comfortable and convenient environment, allowing them to talk freely and in detail (Davis & Craven, 2016). Interviews lasted 20–90 minutes. Each participant was offered a $20 gift card for a local retailer.

### Data Analysis

Participant characteristics were reported using descriptive statistics. Audio recordings were transcribed verbatim and data were de-identified using pseudonyms. After being reviewed for accuracy, transcribed interviews and field notes were imported into MAXQDA software (Version 18.2.0; VERBI GmbH, Berlin, Germany) for data management and analysis. To add reliability and reduce risk of researcher bias, A. W. B. included a Black PhD candidate trained in qualitative research to serve as second reader and coder. A. W. B. used thematic analysis to deductively analyze transcripts that combined inductive coding (to identify emergent themes) and created thematic maps (to group themes; Cormack et al., 2018). Trustworthiness was achieved using the following techniques: Pilot testing the interview guides, keeping detailed field notes, peer debriefing, maintaining a research reflexivity journal, member checking, utilizing multiple data coders and clarification of bias to ensure accuracy (Creswell & Miller, 2000).

## Results

### Participant Characteristics

Fifteen African American family dyads/triads (*n* = 5 G1s, *n* = 15 G2s and *n* = 15 G3s) were interviewed. Nine families were dyads and six were triads. All participants ranged from 24–80 years, the majority (57%) were married, and all had a high school diploma or higher. G1s’ mean age (range) was 71.6 (64–80 years) and parity was 3.2 live births. G2s’ mean age (range) and parity was 53.6 (36–67 years) and 3.6 live births, respectively. G3s’ mean age (range) and parity was 30.6 (24–34 years) 1.9 live births, respectively. See Table 1 for additional participant characteristics.

**Table 1:** Participants’ Characteristics (*N* = 35).

Pseudonym	Generation	Age group (yrs.)	Breastfed	Parity
Louise	G1	64-80	No	3
Martha	G1	64-80	Yes	3
Barbara	G1	64-80	No	4
Sandra	G1	64-80	No	3
Vivian	G1	64-80	No	3
Sherry	G2	36-67	No	2
Sharon	G2	36-67	No	3
Vanessa	G2	36-67	Yes	2
Betty	G2	36-67	Yes	2
Shirley	G2	36-67	No	1
Valerie	G2	36-67	Yes	3
Jennifer	G2	36-67	Yes	3
Yolanda	G2	36-67	No	2
Gloria	G2	36-67	No	5
Karla	G2	36-67	No	1
Pamela	G2	36-67	Yes	4
Andrea	G2	36-67	Yes	3
Roxanne	G2	36-67	No	3
Helen	G2	36-67	Yes	3
Sabrina	G2	36-67	Yes	3
Brianna	G3	24-35	Yes	1
Stacey	G3	24-35	Yes	1
Amber	G3	24-35	Yes	2
Asia	G3	24-35	Yes	2
Katrina	G3	24-35	Yes	1
Dominique	G3	24-35	Yes	3
Lashonda	G3	24-35	Yes	4
LaKisha	G3	24-35	Yes	1
Tiffany	G3	24-35	Yes	2
Tonya	G3	24-35	Yes	2
Kimberly	G3	24-35	Yes	3
Charlene	G3	24-35	Yes	1
Tyesha	G3	24-35	Yes	3
Latoya	G3	24-35	Yes	2
Rhonda	G3	24-35	Yes	1

*Note*. G3 = youngest generation; G2 = youngest generation’s mother/mother figure; G1 = youngest generation’s maternal grandmother/grandmother figure.

Breastfed was defined as feeding mother’s own milk to at least one child for 3 months or more.

### Contextualizing African American Families

#### Self-Defined Role in Family

**Figure 1:**
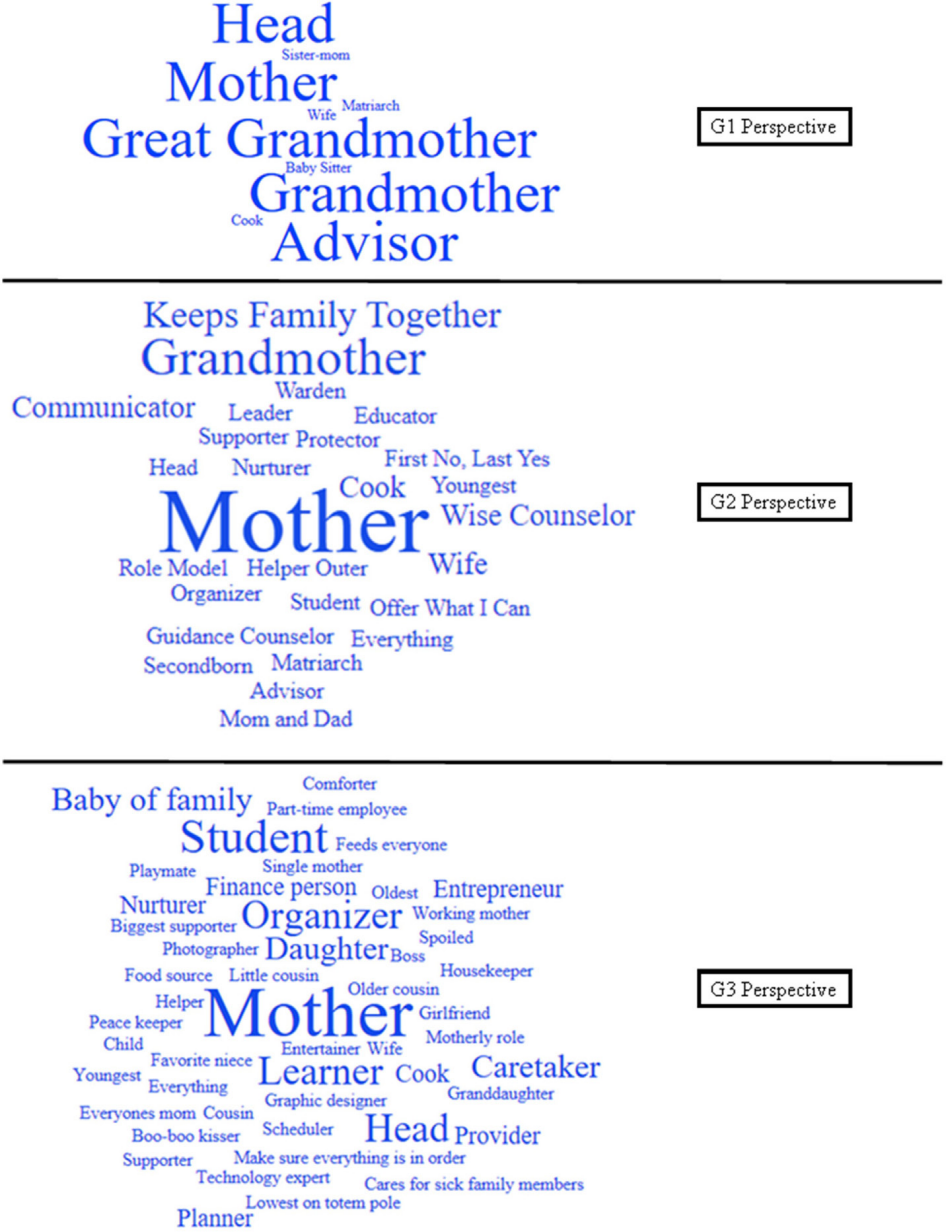
Word Clouds Displaying Each Generation’s Self-Defined Familial Roles.

Exploring the perceived role each generation played in their family provided contextual information to understand the meaning they ascribed to sharing infant feeding information within their family. Each participant discussed their familial role, and Figure 1 displays a word cloud of each generation’s responses. The larger the word or phrase, the more often participants stated it. G1s described their role as “Head,” “Mother,” “Grandmother,” “Great grandmother,” and “Advisor.” G2s described their role as “Keeps family together,” “Mother,” “Counselor,” and “Communicator.” Finally, G3s were careful to clarify to what context they were referring. They would often say in their immediate family, they were “Head,” “Organizer,” and “Provider,” but, in their extended family, they were the “Student,” “Learner,” and “Baby of the family.” Each word cloud became more intricate and complex with each subsequent generation.

### Meaning Attributed to Communicating Infant Feeding Information

Each generation was prompted to reflect on their family communication regarding infant feeding. The meaning they attributed to communicating infant feeding information was expressed across the following themes: My Responsibility, Bonding Experience, Comforting, She Cared and Gained Wisdom. Themes are defined and described below and in Table 2.

**Table 2:** Emergent Themes, Definitions, Examples, and Generational Identification.

Theme	Definition	Examples	Generation
My Responsibility	The conviction and duty G1s and G2s reported about sharing infant feeding information with G3s.	“We supposed to talk to them…It’s important to talk to the young people, to women and everything. But it’s like when you say it, don’t go in there like a know-it-all. But kind of make them feel comfortable. See it from both sides of the fence.” (Martha, G1)	G1s/G2s
Bonding Experience	The closeness and attachment that G2s described having with G3s during their infant feeding discussions.	“It felt good, you know because we were like bonding, you know. Over something different, you know. So, it felt good. (Sharon, G2)	G2s
Comforting	The tranquility and calmness that G2s experienced because of infant feeding discussions with G3s.	“It was really comforting to know that I had some kind of experience and I could share some things that would make her life a little bit easier. Some things that had passed down from my mom.” (Yolanda, G2)	G2s
She Cared	The trust, confidence, and belief that G2s and G1s cared when having infant feeding discussions with G3s.	“Being young you know and never experiencing it, I just kinda said, well my Mama knows best, and I just went with what she said would be the best for my daughter.” (Asia, G3)	G3s
Gained Wisdom	The value G3s placed on the wisdom expressed through infant feeding conversations with G1s and G2s	“It really meant a lot because you can’t, in this day and age, you can’t pay for that wisdom.” (LaKisha, G3)	G3s

*Note*. Generation referred to which generation contributed to each theme. G3 = youngest generation; G2 = youngest generation’s mother/mother figure; G1 = youngest generation’s maternal grandmother/grandmother figure.

#### Theme: My Responsibility

My Responsibility denoted the conviction and duty G1s/G2s reported regarding sharing infant feeding information with G3s. Overall, they believed elders were responsible for passing knowledge and values down to G3s. Louise discussed the importance of elders teaching younger women about infant feeding, motherhood, and womanhood:

It was very important to share information with [my granddaughter], because the scriptures say that the older women are to teach the younger women how to love their husbands, how to be chaste housewives and how to raise their children. And in today’s society, this is what we are missing. We have information for you to bring you to another level. And if we are not teaching, then our generation line is missing a lot of stuff.

In general, G1s/G2s discussed the joy they experienced from sharing their knowledge. Vivian explained:

It’s good to share your knowledge with somebody you care about. Whether they take it or not, it still makes you feel good to share it. And when you find out that they have taken your advice, you really feel good…’cause you feel like you are here for a purpose to teach or to share.… I think it’s good to pass the information that you done gathered in your life on to the younger people. Because the information is the same. It might be done a different way, but you put the idea in their head of how to do this, that, and the other. And they don’t necessarily have to do it the same way you do it, but you done gave them the idea and the knowledge that this can be done this way. ’Cause I believe in not making stuff harder for yourself.

#### Theme: Bonding Experience

Bonding Experience referred to the closeness G2s described having with G3s regarding their infant feeding discussions. Betty said,

It just kind of deepened our relationship because it was something that I had experienced as a mom and was able to pass on to her. So, it’s something that we could talk about…we could laugh about. I mean because we have something else in our little history that we can talk about.

Additionally, Roxanne expressed, “It meant the world to me because I love my daughter and my grandkids. Teaching her about being a mom and feeding her babies brought us closer to each other.” G2s enjoyed the idea of being able to bond with G3s over a topic that was as intimate as feeding children.

#### Theme: Comforting

Comforting represented the tranquility and calmness G2s experienced because of their infant feeding discussions with G3s. Valerie said:

It gave me a sense of security that everything would be OK. Because I’m making sure [my daughter] knows what to look for and what to do.... [A]s a Mom, it’s not like you’re gonna always be there. So, you want to make sure they know.

Additionally, Yolanda said, “It was really comforting to know that I had some kind of experience and I could share some things that would make her life a little bit easier…some things that had passed down from my mom.”

#### Theme: She Cared

She Cared referred to the trust, confidence, and belief G3s described regarding the infant feeding discussions they shared with G1s/G2s. This theme encompassed G3s’ sentiments that G1s/G2s cared for them because they took time to share infant feeding information with them. G3s described three main reasons why they perceived older generations cared: (1) G1s/G2s would not intentionally tell them anything wrong; (2) G1s/G2s gave a personal touch while sharing infant feeding information; and (3) G1s/G2s only wanted the best for them and their children. Asia recalled valuing her mother’s advice:

Being young and never experiencing [breastfeeding], I just kinda said, well my momma knows best, and I just went with what she said would be the best for my daughter. It meant a lot because I was kind of going into the situation blind and young and inexperienced. So, you know I kinda felt like my momma had my back and she wouldn’t steer me wrong.

Additionally, Dominique recalled the hospital being very clinical, but her mother gave something more:

It really meant a lot to have somebody that cares…not that nurses don’t care…some of them do, but some of them will definitely rush you out. “Is the baby breathing? Are you breathing? Good.” And they are gone out of the room.... [I]f I did not have [my mom] helping me with feeding the baby, I wouldn’t have known what to do…she just gave that personal touch.

For G3s that were first-generation breastfeeders (first in their family to breastfeed), they shared that even though older generations may have initially been critical of their breastfeeding decision, they understood their response came from a caring place. Brianna said:

I think when my [mom and grandma] don’t understand something, they…shun it away. Or they say that that’s something that you shouldn’t do. But I know they are only doing and saying that because they only want to see the best for me…I know they still care, they just don’t know how to show that support that’s needed.

#### Theme: Gained Wisdom

Gained Wisdom revealed the value G3s placed on the wisdom expressed through infant feeding discussions with G1/G2s. G3s gave two main reasons why they valued the wisdom they gained: (1) G1s/G2s had experienced motherhood before and (2) the wisdom from their elders is priceless. Kimberly listened to her mother’s instructions because of her prior motherhood experience:

I felt she knew what was going to be best. She’s been down this road before. I feel like why not listen to her.…And she was like, “Okay, you’re going to breastfeed,” though I had not made up my mind. So, I’m thinking in my head she told me I’m gonna breastfeed. Maybe this is what I need to do….I’ll look more into it.

Amber discussed the importance of recognizing and honoring the wisdom that elders contribute, some of which was nonverbal,

Well, it was more so like with your mother, or your grandmother, or your aunt, it’s not always…a real conversation. It’s more like they do this to your baby…you just go with it. You don’t tell them “no.” And I think it’s funny ’cause I think as Black women, the more educated we get, the more we get away from letting our elders do what they know to do. ’Cause obviously what they know has worked. So, it was more like they tell you, “This is what you need to do. You need to try this. You need to try that.” Oh Okay. Yeah, I’m on it. I’ll do that.

LaKisha lauded her grandmother for the wisdom she shared: “It really meant a lot because you can’t, in this day and age, pay for that wisdom...I feel I was very blessed to have [my grandmother].” G3s generally described trust and acceptance toward the infant feeding information shared within their family.

## Discussion

Our findings may be used to strengthen understanding of the interplay of intergenerational familial roles and meaning ascribed to communicating infant feeding information across three generations of African American women. We have added to the literature that using family-centered approaches to breastfeeding promotion and support may be beneficial for African American families, as this racial group tends to be collectivistic (kinship-centered) rather than individualistic (Steers et al., 2019). These findings have extensive implications for clinicians, educators and scholars who work with African American families and have the potential to contribute to achieving health equity in this community. Novel findings were: (1) Older generations (G1s/G2s) described a moral responsibility to communicate information with younger generations, which includes topics of infant feeding and beyond; (2) G2s described comfort and a strong bond from communicating infant feeding information with the younger generation (G3s); and (3) younger generations described trust and acceptance of the infant feeding information and wisdom they received from older generations.

G1s/G2s discussed the symbolic meaning of moral responsibility related to passing on knowledge and wisdom to the next generation, which is a consistent theme throughout African American history (Bronner, 1998; Hecht et al., 2002; Osei-Boadu, 1990). African American women and mothers have long used teachings as a form of protection for their children, as an act of maternal love, and as a central principle of their motherwork (Collins, 2005; McLoyd et al., 2019). Our findings also align with generativity concepts, which include concern and need to nurture and guide younger generations (Ashida & Schafer, 2015; Fabius, 2016). Older generations tended to define their familial role as “head,” “advisor,” “communicator,” and “counselor,” which may help to explain their conviction to share infant feeding information with G3s. Therefore, those who work with African American families should consider integrating concepts of generativity to strengthen and enrich their breastfeeding support efforts. Leveraging the social influence from older generations and including them in infant feeding conversations at prenatal or well-baby visits, and in educational programs, would honor the role of older generations in African American families and provide cross-generational influence.

In addition to responsibility, G2s reported bonding over something new in their mother–daughter relationship, as well as feeling comfort knowing they shared information that G3s could use. They described serenity in knowing that G3s gained knowledge and wisdom about motherhood, womanhood, and other aspects of life. This finding contributes to further understanding the dynamics of familial roles among African Americans. Bonding has been associated with trust, and positively affects overall self-esteem in African Americans (Causey et al., 2015). As mentioned earlier, mothering while Black requires constant concern for protection that includes various socialization strategies (Malone Gonzalez, 2020 ). Future breast/chestfeeding promotion efforts may benefit from reframing our current approach to including protection language and not solely support language. Proper messaging is the crux of breastfeeding promotion, support, and protection. For example, we could educate older generations about the importance of encouraging breastfeeding, which may increase the chances that information funnels down to the younger generation; thereby acting as another method of protection. To effectively bring older generations into infant feeding conversations, lactation professionals must first recognize, honor, and respect the grandmother role, and understand the value each generation places on shared infant feeding information within African American families.

G3s indicated a high level of reverence for G1s/G2s, which is a cultural tradition placing value on respecting and obeying elders (McLoyd et al., 2019). G3s demonstrated this reverence in the nuanced way they defined their familial roles—being the head of their household, while also recognizing that they were students and learners in their extended families. G3s generally described trust and acceptance of the infant feeding information shared by G1s/G2s. Considering that African Americans experience some level of medical mistrust (Jaiswal, 2019), understandably G3s found G1s/G2s to be a trusting source of information. G3s described the wisdom they gained from G1s/G2s, and that G1s/G2s cared because of their willingness to share their infant feeding knowledge and stories. Feeling cared for contributes to a mother’s sense of overall well-being (Miller & Wilkes, 2015). African American communities disproportionately report experiencing substandard maternity care and do not feel cared for by the medical community (Robinson et al., 2019). Frankly, in the United States, African American women are three to four times more likely to die from pregnancy or childbirth-related reasons (Centers for Disease Control and Prevention, 2017) because of interlocking systems of oppression, including the lack of value placed on their lives within the U.S. healthcare system. Additionally, various researchers have demonstrated that healthcare providers offer breastfeeding advice, education, and support less often to African Americans than other racial/ethnic groups (Asiodu et al., 2017; Davis, 2019; Johnson et al., 2016). Providers should recognize that this generation associates receiving infant feeding information with feelings of care and concern and provide them with equitable breastfeeding education.

Interventions aimed to increase informal breastfeeding support are likely to increase breastfeeding rates (DeVane-Johnson et al., 2018). Since African Americans tend to identify with collectivism, there are direct implications for public health programs and interventions. The following should be considered during the design phase: (1) Reverence for the role and authority of elders, and cultural traditions are foundational values (McLoyd et al., 2019); (2) elders are vital in conveying information to younger generations (McLoyd et al., 2019); (3) multigenerational and extended families influence beliefs of individuals within the family (Fabius, 2016); (4) intergenerational interactions and communication are key (Fabius, 2016); and (5) the community-based participatory research model is an important element for successful interventions in addressing minority health disparities (National Academies of Sciences, Engineering, and Medicine, 2017).

### Limitations

In qualitative research, participants and researchers engage directly, which not only encourages prolific discussion and thick descriptions, but also can increase the possibility of researcher bias. Additionally, social desirability bias may have influenced participants’ responses. While every effort was made to develop rapport with the participants and to elicit accurate responses, A. W. B. asked intimate questions about their familial relationship status and the meaning ascribed to sharing infant feeding information, and participants may have felt uncomfortable answering accurately.

## Conclusion

This novel study provides unique perspectives to existing infant feeding literature as few researchers have examined the interplay of self-defined familial roles and how three generations ascribe meaning (value) to shared infant feeding information within African American families. Our findings suggested potentially unexpected pathways to increasing health equity through recognizing and supporting the strengths and resource-richness of intergenerational infant feeding communication within African American families using strength-based, empowerment-oriented, and ethnically sensitive approaches. The meaning examined may provide a framework for further exploration of grandmothers’ roles in breast/chestfeeding support, and the specific contexts under which this may occur. Providing equitable care to African American families means respecting each generation, gauging their feeding attitudes, meeting them where they are, and listening to them.
